# Calcium Intake and Food Sources Among Children, Adolescents and Women in Madagascar: Results from a Nationally Representative Survey

**DOI:** 10.3390/nu18071041

**Published:** 2026-03-25

**Authors:** Lantonirina Ravaoarisoa, Valeria Galetti, Ravakamaharitra Rakotovao, James Peter Wirth, Carla El-Mallah, Fabian Rohner, Mathieu Joyeux, Niry Randrenarizo, Zeinab Annan, Malaza Armel Alex Razanatsila, John Syllie Noela Randriarivony, Zo Nantenaina Raveloson, Rita Wegmüller

**Affiliations:** 1 CAETIC Développement, Antananarivo 101, Madagascar; lantonirinadr@yahoo.fr (L.R.); caetic.dev@gmail.com (R.R.); niry.randrenarizo@caetic.mg (N.R.); 2 Faculté de Médicine, Université d’Antananarivo, Antananarivo 101, Madagascar; razanatsilaarmel@gmail.com; 3 GroundWork, 7306 Fläsch, Switzerland; valeria@groundworkhealth.org (V.G.); james@groundworkhealth.org (J.P.W.); carla@groundworkhealth.org (C.E.-M.); fabian@groundworkhealth.org (F.R.); 4 United Nations Children’s Fund (UNICEF), Antananarivo 101, Madagascar; mjoyeux@unicef.org (M.J.); zeiannan05@gmail.com (Z.A.); 5 Gesellschaft für Internationale Zusammenarbeit (GIZ), Antananarivo 101, Madagascar; john.randrianarivony@giz.de; 6 Service de Nutrition, Ministère de la Santé Publique, Antananarivo 101, Madagascar; nantenainarz@yahoo.fr

**Keywords:** calcium intake, calcium food sources, inadequate intake, Madagascar, preschool children, adolescents, women of reproductive age, public health

## Abstract

**Background**: Many countries in sub-Saharan Africa are at risk of inadequate calcium intake, yet no data exist for vulnerable population groups in Madagascar. We aimed to assess daily calcium intake, the major contributing food sources, and the prevalence of inadequate intake in young children, adolescents, and women of reproductive age. **Methods**: The 2024 National Micronutrient Survey used a two-stage probabilistic design across all 23 regions. The daily calcium intake was estimated using a food frequency questionnaire that focused on calcium-rich foods that are commonly consumed in Madagascar and the calcium concentration measured in drinking water. **Results**: Calcium intake was low across all population groups, averaging 200–300 mg/d in adolescents and women and below 180 mg/d in young children. The prevalence of inadequate intake exceeded 96% in every population group. While calcium intake increased with increasing household wealth in children, the opposite pattern was observed for adolescents and women, whose intake decreased with increasing wealth. The main contributors to calcium intake were cassava leaves, cassava roots, small fresh and dried fish eaten with bones, drinking water across all population groups, and breastmilk in young children. **Conclusions**: The calcium intake is low throughout Madagascar and across all demographic groups. Strategies to improve intake are urgently needed and should include promoting continued breastfeeding and the consumption of calcium-rich, locally available, affordable foods such as small fish eaten with bones and leafy green vegetables, alongside a consideration of wheat flour fortified with calcium.

## 1. Introduction

Calcium is an essential mineral that serves as a major structural component of bones and plays a critical role in maintaining skeletal strength [1]. An adequate calcium intake during childhood and adolescence is necessary to achieve the optimal peak bone mass, whereas maintaining sufficient intake in adulthood helps prevent age-related bone loss [2]. Consequently, an inadequate calcium intake is associated with rickets and impaired growth in children [3], osteoporosis and elevated fracture risk in adults [4], and an increased risk of pre-eclampsia during pregnancy [5].

Globally, approximately 46% of the population consumes diets that are low in calcium, with substantially higher prevalences in central sub-Saharan Africa (88%) and South Asia (83%) [6]. Recent modeling analyses confirm that the diet inadequacy remains extremely high in sub-Saharan Africa, particularly among adolescents aged 10–20 years [7]. Within this regional context, Madagascar appears among the countries with the highest estimated prevalence of inadequate calcium intake, approaching 90% [7], which is consistent with the earlier analyses that are based on national food availability data [8,9,10].

In the absence of an established biomarker for calcium status, dietary assessment methods remain the primary approach to estimating intake adequacy among specific population groups [11]. Although the international consensus on the average calcium requirements has not been fully established, many countries adopt a reference value of approximately 800 mg/d for adult males and for nonpregnant, nonlactating females [12]. The average dietary calcium intake varies markedly across countries: a 2017 systematic review reported a range from 175 mg/day in Nepal to 1233 mg/day in Iceland [13]. While data were not available in many countries in Africa, those countries with data indicate moderately low intakes, typically between 300 and 700 mg/day.

Despite evidence of potentially widespread inadequacy, dietary intake data for key population groups in Madagascar, particularly those most vulnerable to malnutrition, remain unavailable. Generating such data is essential for guiding nutritional policy and informing the design of targeted interventions.

Therefore, the aim of the present study was to estimate the daily dietary calcium intake, to identify the main contributing food sources, and to determine the prevalence of inadequate intake among preschool children, adolescents, and women of reproductive age in Madagascar.

## 2. Materials and Methods

### 2.1. Study Design and Participants

The population’s calcium intake was estimated as part of the national survey on micronutrient deficiencies in Madagascar (enquête nationale sur les carences en micronutriments à Madagascar—ENCM) [14], which was conducted in 2024. The study was a national cross-sectional stratified survey based on a probability sample with 23 strata and a 2-staged sampling approach. The 23 regions of the country served as the strata. In the first stage, the primary sampling units were formed by fokontany, the smallest administrative units in Madagascar. The fokontany were randomly selected with a probability that was proportional to the population size. A total of 368 fokontany (16 per stratum) were selected. In the second stage, 10 households were randomly selected with an equal probability of being chosen from a complete household listing. All the households living in Madagascar during the time of the survey were eligible for participation. All the members of the selected households belonging to the target groups (preschool children 6–59 months, adolescents 10–19 years, and women 15–49 years) and meeting the eligibility criteria (consenting to participate and considered a household member by the head of the household) were recruited. The nonpregnant females aged 15–19 years were allocated to two different groups during the data analysis: adolescents and nonpregnant women.

The survey protocol was approved by the Comité d’Éthique de la Recherche BioMédicale (CERBM), the Biomedical Research Ethics Committee of the Ministry of Public Health (N°045-MSANP/SG/AMM/CERBM), and it was registered with Open Science Framework (https://osf.io/kuywa/files/osfstorage, accessed on 11 February 2026).

### 2.2. Data Collection

The data collection was conducted from 10 June to 7 September 2024. The data collection was carried out by 12 teams across the country, with each team having two interviewers. The interviewers were trained in the introduction of the pre-tested questionnaires and on all collection procedures. The questionnaires were available in French and Malagasy. The household questionnaire covered the demographic, socioeconomic, and food security data and was administered to the household head, the spouse, or both, upon oral consent. Individual questionnaires covered information on health and calcium intake and were administered after obtaining written informed consent from all adults and parental written consent for all children and adolescents who were < 18 years and assent for all adolescents who were 10–17 years.

The calcium intake was estimated using a food frequency questionnaire (FFQ) with a 7-day recall period (https://osf.io/kuywa/files/osfstorage, accessed on 11 February 2026) for all the individuals selected from a household. This approach was designed to capture the usual intake of calcium-rich foods rather than short-term consumption, which is consistent with the study objective. The list of calcium-rich foods used in the FFQ was developed specifically for the context of Madagascar. To identify calcium-rich foods, food composition tables for Madagascar, Malawi, Mozambique and the FAO/INFOODS Food Composition Table for Western Africa were consulted [15,16,17,18]. This list was then reduced through expert consultation to include only foods that were consumed regularly and in sufficiently large quantities to make a potentially significant contribution to calcium intake (see Supplementary Table S1 for the list of foods included). The portion sizes were estimated using a picture catalogue illustrating the standard portion sizes for all food items included in the FFQ. For foods with discrete units (e.g., a baguette), images showed portions such as half a baguette, whereas for other foods, portion sizes were represented using commonly used kitchen utensils (e.g., bowls). The corresponding utensils were also carried by interviewers and shown to participants during the interview to aid their portion size estimation. In addition, the calcium content of drinking water was assessed at the cluster level to take into account the intake from drinking water. To execute this, in each cluster, one water sample was taken from a supply point regularly used as a source of drinking water by the residents for semi-quantitative calcium analysis on site using a rapid titrimetric test (Visocolor alpha Carbonate hardness titrimetric test kit, Macherey-Nagel, Oensingen, Switzerland).

The computer-assisted personal interview approach was adopted, allowing the use of tablets for direct data entry during the field collection. This application was developed by combining two software programs: the first application, developed on CS Entry (version 7.7.3; US Census Bureau, Washington, DC, USA), was used for the enumeration and random selection of households, and the second application, constituting the electronic questionnaires, developed on KoboCollect (v1.25.1, Cambridge, MA, USA), was used to collect information from the household and individual levels.

### 2.3. Validation of FFQ Results

Since there is no reliable calcium biomarker to compare the FFQ method to, we compared it to the existing dietary intake data that was available. However, such data is only available at the national level and not at the level of different population groups that were seen in our study. Therefore, we additionally used FAO’s estimated calcium availability from their supply utilization accounts (SUA) [19] in combination with the adult male equivalent (AME) method [20,21] to judge the accuracy of our data at the population group level. We used the most recent SUA estimates for calcium availability for 2022, which is 268 mg/capita/day. As per capita intake is calculated by the food available in a given year divided by the overall population, this allowed us to calculate the overall calcium availability from foods (not including water) for 2022. In order to take into account the different energy needs of different population groups, we used the AME approach [20,21], considering the whole population of Madagascar as the household to assign specific portions of the total calcium available to each population group. Within each population group, we then divided the portion by the number of people within that population group to get a per capita intake for that specific population group. Since the SUA data was based on data from 2022, we used the overall population estimate used by FAOSTAT, which is based on the World Bank population estimates for that year, as well as the demographic data from the 2018 population census to extract the AME values for each population group. We then compared these estimates with the results that were obtained in our survey. This allowed us to assess whether choosing the calcium-rich food groups in the FFQ in our survey would result in comparable estimates to the SUA, as the SUA takes into account all the food groups.

### 2.4. Calculation of Calcium Intake and Statistical Analysis

The data analysis was performed using StataNow/SE version 19.5 (Stata Corp., College Station, TX, USA).

The weekly consumption of each calcium-rich food from the FFQ was multiplied by its content of calcium (Supplementary Table S1). This value was then divided by seven to determine the daily calcium consumption from each food. The individual daily calcium intake was calculated by combining the intake from the different calcium-rich foods, the intake from drinking water, and the intake from breastmilk in children aged 6–23 months old. The quantity of water and breastmilk consumed was not assessed for each individual but was based on data from the literature [22,23,24,25] (Supplementary Table S2). The intake from breastmilk was only included if, during the interview, the caregiver responded that the child was still breastfed. The calculated daily calcium intake was compared with the estimated average requirements (EAR) from the Institute of Medicine [12] for each population group and each specific age sub-group (Supplementary Table S2).

The daily calcium intake was calculated for each population group overall (children aged 6–59 months, adolescents aged 10–19 years, and nonpregnant and pregnant women of reproductive age) and by several socio-demographic characteristics. The household wealth was assessed by the Demographic and Health Surveys Wealth Index and the food insecurity by the Food and Nutrition Technical Assistance Household Food Insecurity Access Scale. The sampling weights were applied to account for the unequal probability of selection, and 95% confidence intervals (CI) were calculated to reflect the complex sampling design. Pairwise deletion was applied to missing data, and all available observations were retained for each specific analysis. The sub-group differences were assessed using a one-way ANOVA. For readability and conciseness, the prefix “Grand” for the different zones has been omitted throughout the manuscript; the zones are therefore referred to, in their English translations, simply as North, Center, East, West, and South throughout the text.

The geospatial analysis was used to map the calcium intake, expressed as the percentage of the EAR from the total diet (including drinking water and breastfeeding for young children) and from drinking water alone. The cluster-level mean percentages were calculated and interpolated using inverse distance weighting. A power parameter (*p*) of 4 was applied to reduce the influence of distant clusters, given the large spatial separation between some sampling locations. The analyses were performed in QGIS (version 3.4.1).

## 3. Results

### 3.1. Response Rates and Demographic Characteristics

In total, 3658 households of the 3707 eligible households were interviewed (98%). In these households, individual interviews were conducted in 2168 children who were 6–59 months of age (96% of eligible children), in 3178 adolescent boys and girls who were 10–19 years of age (86%), in 3399 nonpregnant women of reproductive age (93%), and in 283 pregnant women (97%). The nonpregnant females between 15 and 19 years were counted in the adolescent population group, as well as in the nonpregnant women of reproductive age (WRA) group.

The full descriptives of the included households and the socio-demographic and health characteristics of the investigated population groups are available from the ENCM report [14].

In brief, almost 80% of the households included were in rural areas. In 76% of the households, the head of the household was a man. The households counted on average 4.4 household members. Almost all households (99%) used solid fuel for cooking, whereas 18% used electricity as a source of domestic lighting and 19% used solar energy. Overall, 78% of the households were food insecure, with 35% being severely food insecure, 26% being moderately food insecure, and 16% having a mild food insecurity. Close to two-thirds (60%) of households consumed safe drinking water, and only 10% of the households had adequate sanitation, as those with improved sanitation mostly shared toilets with other households, and open defecation was practiced by 38% of the households.

The gender distribution (50.2% male) as well as the distribution between age sub-groups was balanced in the 2168 preschool children. Close to 50% of children showed some sort of inflammation (elevated C-reactive protein or α1-acid-glycoprotein in their blood), and 36%, 19% and 14% indicated a fever, diarrhea or a lower respiratory tract infection, respectively, in the two weeks preceding the survey. A positive malaria rapid diagnostic test was found in 8% of the children, and 21% met the minimum dietary diversity requirement according to the definition of the WHO and UNICEF Joint Monitoring Program [26].

Slightly more than half of the 3178 adolescents were females (52.9%). Of the adolescent girls, 56% were post-menarche. The infections in the past 2 weeks were less common in this age group compared to that of younger children (78% showed no sign of inflammation), but 22%, 7% and 6% reported having had an episode of fever, diarrhea or lower respiratory tract infection, respectively, in the past two weeks. The prevalence of a positive rapid diagnostic test for malaria was 11%, and 23% met the minimum dietary diversity.

Of the 3399 nonpregnant WRA, 57% were married, and 28% were breastfeeding at the time of the survey. Around one-fifth of the women showed any sign of inflammation, and 4% tested positive for malaria. The minimum dietary diversity was met by 25% and was strongly associated with household wealth and food security.

Around two-thirds (65%) of the 283 pregnant women were younger than 30 years, and 40% were in the second trimester of pregnancy.

### 3.2. Appropriateness of the Calcium Intake Assessment Method

**Figure 1:**
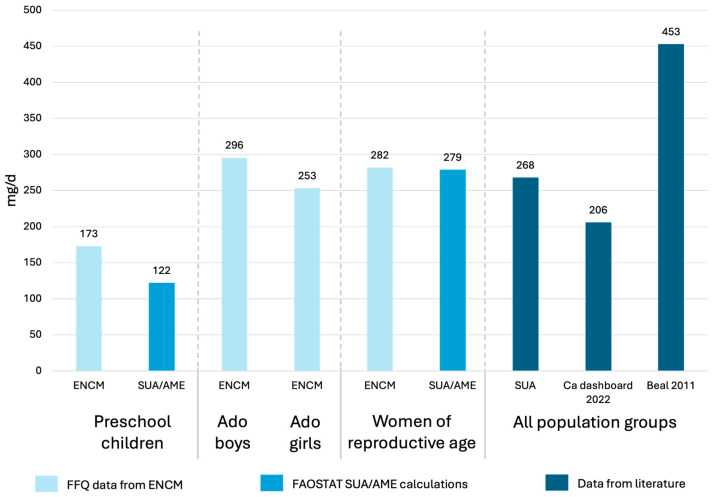
The comparison of the mean calcium intake (mg/d) from the FFQ results collected in the ENCM with results calculated from FAO’s SUA and applying the AME method, and with estimates from the literature for different population groups in Madagascar. AME, adult male equivalent; ENCM, enquête nationale sur les carences en micronutriments; FFQ, food frequency questionnaire; SUA, supply utilization accounts.

To check the validity of the 7-day food frequency questionnaire (FFQ) targeted to calcium-rich food groups in combination with a picture catalogue to estimate portion sizes, we compared our results to (1) apparent intake estimates for the whole population from the literature, and (2) to FAO’s SUA data, applying the AME method to get population-specific estimates. Figure 1 shows that the estimates from our FFQ were higher in young children (173 vs. 122 mg/d) but similar in WRA (282 vs. 279 mg/d) compared to using FAO’s SUA and applying the AME. The discrepancy in children is likely due to the calcium from drinking water and breastmilk, which is not included in the FAO’s SUA calculations. When comparing our estimates in women and adolescents with national apparent intake estimates, our estimates are slightly higher when compared to those of the Calcium Dashboard from 2022 [10] but considerably lower than older estimates from 2011 [8]. Overall, the results obtained by the FFQ seem plausible.

### 3.3. Calcium Intake, Risk of Inadequate Intake, and the Association with Socio-Demographic Factors

Table 1 shows the estimated daily calcium intake, including drinking water and breastmilk in children <24 months old, and the risk of inadequate intake in the different population groups assessed within the ENCM. The median and mean calcium intake were low in all population groups, with estimates below 180 mg/d for young children and around 200 to 300 mg for all the other population groups. The risk of inadequate calcium intake was high and above 96% in all population groups. Due to the low number of pregnant women, no sub-group analyses were conducted.

The calcium intake in the different population groups differed by several socio-demographic characteristics (Table 2). Male children and adolescents showed higher intakes than their female counterparts. The nonpregnant WRA who were breastfeeding at the time of the survey had a higher calcium intake than those who were not breastfeeding. There were differences in the household food insecurity in children and adolescents, with no clear trend in either population group. The calcium intake differed by the household’s wealth in all population groups, with a clear pattern in adolescents and nonpregnant WRA, in whom calcium intake decreased with increasing wealth. The calcium intake was also lower among adolescents and nonpregnant women in urban than rural areas, where households tend to be concentrated in higher wealth quintiles. Furthermore, there were differences in intake across all population groups by zone, with the North and the South showing the highest intakes.

### 3.4. Contribution of Different Foods and Drinking Water to Calcium Intake

**Figure 2:**
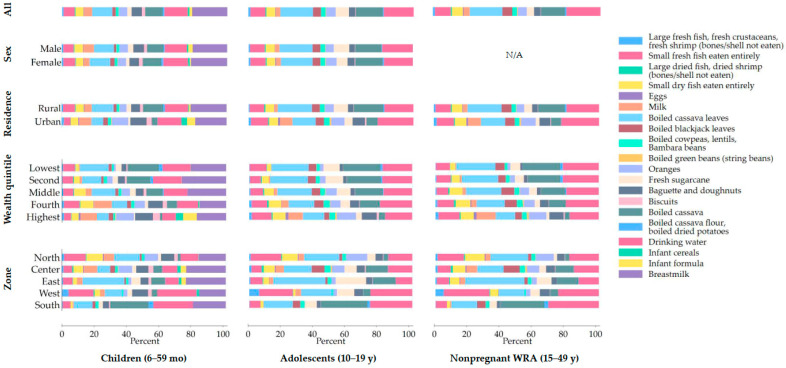
The foods contributing to the daily calcium intake overall, by sex, residence, wealth quintile, and zone in the different population groups. WRA, women of reproductive age.

The foods contributing most to the calcium intake were similar in all population groups: boiled cassava leaves, boiled cassava roots, small fresh and dried fish eaten with bones, drinking water, and, additionally, breastmilk in young children (Figure 2). Breastmilk contributed 21% to the overall calcium intake in children; however, the contribution was only estimated for children who were 6–23 months old, in whom it was around 50%. The boiled cassava leaves contributed around 16–20% (corresponding to 55 mg/d in nonpregnant WRA) and boiled cassava around 15% (corresponding to 40 mg/d in nonpregnant WRA) to the overall calcium intake in all population groups. Overall, the contribution of drinking water was also relatively high in all population groups, with around 19% in adolescents and nonpregnant WRA and around 14% in children, contributing most in nonpregnant WRA with 57 mg/d. The contribution from small fresh fish and small dried fish together, both consumed with bones, was also around 14% in all population groups. Infant cereals and infant formula, which are potential additional calcium sources for young children, only contributed around 3% (corresponding to <5 mg/d).

**Figure 3:**
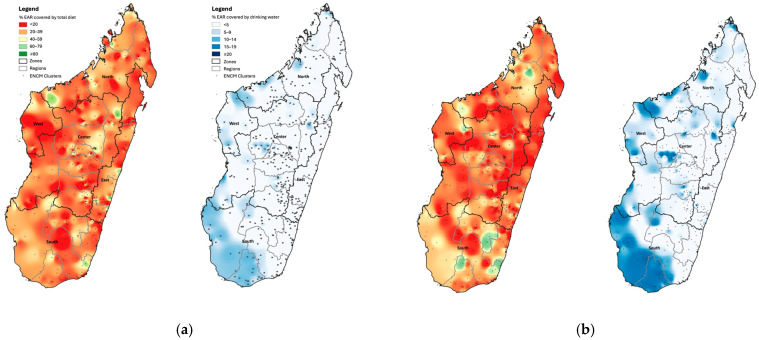

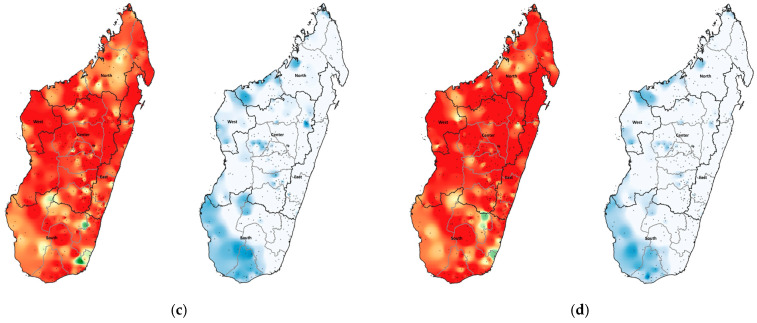
The percentage of the calcium EAR from the total diet including drinking water (red maps) and from drinking water alone (blue maps) in (**a**) young children aged 6–59 mo; (**b**) nonpregnant women of reproductive age, aged 15–49 y; (**c**) adolescent boys aged 10–19 y; and (**d**) adolescent girls aged 10–19 y. EAR, estimated average requirement.

While the differences in foods that were contributing to the calcium intake were small in relation to sex in children and adolescents, differences by residence, wealth quintile, or zone were larger for all population groups (Figure 2). There were remarkable differences in relation to the foods contributing to the calcium intake between children from rural and urban areas. The intake from infant formula and infant cereals was around six times higher in urban than rural children, with a contribution to the overall calcium intake of close to 10% in urban areas, while this was below 2% in rural areas. The children living in rural areas showed higher contributions from boiled cassava leaves and cassava, as well as from the small fresh and small dried fish eaten with bones. The boiled cassava leaves and cassava, along with sugarcane, also contributed more to the calcium intake in adolescents and nonpregnant women living in rural areas than those living in urban areas, while the contribution from small fresh and small dried fish eaten with bones, milk, oranges, and baguettes/doughnuts was higher in those living in urban areas. The patterns in relation to the wealth quintile were similar in adolescents and nonpregnant WRA, with the contribution from fish and seafood products, milk, oranges, and baguettes/doughnuts increasing with increasing wealth and the contribution from cassava leaves, cassava, and fresh sugarcane decreasing. The pattern in young children was similar, but the most remarkable difference was seen in the children from the highest wealth quintile, where close to 15% of their daily intake was consumed through infant cereals and infant formula, while this contribution was negligible in all other wealth quintiles. Furthermore, there were some differences in the consumption patterns in relation to the zones, with the South showing a higher contribution to overall calcium intake from boiled cassava than any other zone in all the population groups. In the East zone, the contribution from cassava leaves was higher compared to the other zones, while milk was more important in the Center, and fish and seafood products were more important in the West and North. The contribution from drinking water was lower in the East compared to the other zones, which is in line with the lower water calcium concentrations seen in that region (Figure 3).

Most food groups were significantly positively associated with the total calcium intake, as shown by the multivariable linear regression analysis that accounted for age, sex, residence, zone, and wealth quintile and took into account the survey design (Supplementary Table S3). Five food groups of those we selected as high-calcium foods were not associated with the total calcium intake in any of the population groups: large dried fish and dried shrimps consumed without bones or shell; eggs; boiled cowpeas, lentils, and Bambara beans; and boiled green beans and biscuits. Furthermore, the food group of “baguettes, doughnuts” was not associated with the total calcium intake in adolescents and nonpregnant WRA, and the group of boiled “cassava flour, boiled dried potatoes” was not associated with the total intake in adolescents. These results highlight that most of the assessed food groups in the FFQ were associated with the total calcium intake, including drinking water and breastmilk in young children.

### 3.5. Geographical Distribution of the Total Calcium Intake and the Calcium from Drinking Water

There are geographical differences in the overall calcium intake and also in the calcium intake from drinking water, as this largely depends on the water’s calcium content. The maps in Figure 3 show the geographical distribution of the percentage of the EAR that is covered by total calcium ingested from the diet, including drinking water, and breastmilk in children, and the percentage of the EAR that is covered from drinking water alone. The first map for each population group shows that less than 20% of the EAR is covered through food and drinking water in most parts of the country, especially for adolescents, with a slightly better situation regarding children and nonpregnant WRA. There are a few pockets that have coverage above 60%, mainly in the southern part of the country, for adolescents and nonpregnant WRA. In general, the calcium intake tends to be higher in the south-western part of Madagascar, where the contribution from drinking water is higher (blue maps), suggesting an important contribution from drinking water.

## 4. Discussion

Our nationally representative survey shows that the calcium intake in Madagascar is extremely low in all the population groups that were assessed (young children, adolescents, and nonpregnant and pregnant women), with more than 96% not meeting the EAR. The use of a food frequency questionnaire targeting commonly consumed calcium-rich foods yielded plausible intake estimates when compared with the limited data available in the literature.

Due to the lack of a reliable calcium biomarker, dietary intake methods are typically used to estimate inadequate intake. However, there is no gold standard method. The indirect approaches, such as food balance sheets, rely on secondary data to estimate national food availability, and the per capita nutrient supplies [11]. These approaches do not adequately capture the small-scale agricultural production, the harvest of wild plants, the household food waste, or the distribution of food across demographic groups [7]. In contrast, direct methods, as used in our survey, collect individual-level data, allowing an estimation of actual intake rather than availability [7,11]. Our study used a calcium-specific FFQ with a 7-day recall period based on pre-selected calcium-rich foods that are regularly consumed. Similar FFQs have demonstrated an acceptable to high validity when compared with weighed food records in various U.S. populations, with correlation coefficients ranging from 0.33 to 0.85 [11]. To enhance accuracy, we drew on multiple African food composition tables [15,16,17,18] and publications [27,28] and used a pictorial portion-size catalogue.

In the absence of previously published intake data for different population groups in Madagascar, we compared our estimates with the apparent intake derived from FAO food availability data [19]. Using the AME method to allocate FAO estimates to population sub-groups, we found that calcium intakes were broadly comparable for adolescents and WRA, though slightly higher among young children in our survey. This difference is likely explained by our inclusion of calcium from drinking water and breastmilk, which together contributed approximately one-third of the total intake, narrowing the expected disparities between dietary intake and apparent intake, which typically overestimates the consumption. Similarly, a study from Malawi observed higher micronutrient intakes using a 24 h recall compared with the apparent intake from household consumption and expenditure survey data [29]. Our intake estimates for women of reproductive age and adolescents are also aligned with the national modeled estimates from the Global Calcium Dashboard [10], which are marginally lower, likely due to the exclusion of drinking water as a calcium source. The older FAO-based estimates from 2011 were nearly double our values [8], most likely reflecting their assumption of a fixed drinking water calcium concentration (42 mg/L), which exceeds the mean concentration that was measured in our study (24 mg/L). In that analysis, water contributed to 11% of the national calcium availability, whereas in our study, it contributed to around 20%. Overall, our intake values were very low, with around 180 mg/d in young children and around 200–300 mg in adolescents and women of reproductive age across all geographic regions and socio-demographic groups, but are comparable to estimates from Gambia [30] and Nigeria [31].

The existing national estimates of the proportion of the population with inadequate calcium intake rely primarily on food availability, not consumption. These data suggest that sub-Saharan Africa has among the highest prevalence of inadequate calcium intake. Using 2011 food supply data, the estimated prevalence of inadequate intake in the region was around 80% [8,9], with a modest decline in 2018 estimates (74%) [32]. Using data from the Global Burden of Disease study 2019, similar prevalences between 73 and 88% were estimated for the different regions of sub-Saharan Africa [6]. A more recent modeling analysis combining individual dietary surveys, household surveys, and national food supplies across 185 countries confirmed that the inadequacy remains extremely high in sub-Saharan Africa, particularly among adolescents aged 10–20 years [7]. Madagascar consistently appears among the countries with the highest prevalence: around 90% in this most recent modeling analysis and 75–100% in earlier analyses [8,9,10]. Based on the 2018 data, Madagascar is categorized as having both a high disease burden related to inadequate calcium intake and a low policy environment supporting calcium interventions [10]. The prevalence of an inadequacy exceeding 96% in all population groups in our study strongly suggests that the situation has not improved over the past decade. Similarly, the levels of inadequacy (99%), spread across all socioeconomic levels, have been reported among Nigerian children living in Ibadan [31], whereas adequate intake that was observed in adults in Nairobi [33] likely reflects a substantially higher dairy consumption, a food category that contributed very little to the calcium intake in Madagascar.

Across the population groups, the main contributors to calcium intake in Madagascar were boiled cassava leaves, boiled cassava, small fish (fresh or dried) eaten with bones, and drinking water. Additionally, breastmilk was a major contributor in children, particularly in the younger group of 6–23-month-old children. The calcium intake from drinking water varied widely by zone and was highest in the South and lowest in the East, which was consistent with the regional differences in soil calcium content [14]. In the South, drinking water supplied nearly 30% of the calcium intake among all population groups, compared with about 10% in the East, where soil calcium is the lowest. The high contribution of boiled cassava, particularly in rural areas, helps explain the slightly higher intakes among rural adolescents and nonpregnant women and the relatively higher intake observed in the South, where cassava is a key staple crop [34]. The inverse association between household wealth and calcium intake in adolescents and women likely reflects a lower consumption of traditional calcium-rich staples such as cassava roots and cassava leaves, which were major contributors to the total intake in this setting. In contrast, higher intakes in wealthier children may be driven by a greater consumption of infant formula and infant cereals (Figure 2). These interpretations should be made cautiously, given the lack of a biomarker and the limitations of the dietary assessment. These findings partly mirror the evidence from rural Gambia, where low calcium intake across the life course is linked to a lack of dairy consumption [30], a pattern which is common in many resource-limited settings in Africa.

Given the very low calcium intake that was observed, strategies to increase intake are urgently needed across all population groups and regions. Promoting the consumption of calcium-rich foods that are locally available and affordable, such as small fresh or dried fish consumed with bones and leafy green vegetables (e.g., blackjack and cassava leaves) that have been identified as rich sources of calcium in another study [35], may be effective. Small fish are naturally rich in calcium and have been shown to increase intake and treat rickets [36,37]. Importantly, calcium from small fish that are consumed with bones is relatively well absorbed (approximately 25–35%, which is comparable to the calcium absorbed from dairy products), making them a highly bioavailable source of calcium [38]. However, the impact of interventions promoting small fish consumption may be limited among poorer households, whereas leafy green vegetables are generally affordable across all wealth groups. Although several leafy green vegetables contain substantial amounts of calcium [39], their bioavailability is often low (approximately 5–15%) due to the presence of absorption inhibitors such as oxalates [38,40]. Our findings indicate that cassava leaves are already widely consumed, while blackjack leaves, containing more than twice as much calcium as cassava leaves, are consumed in much smaller quantities across all wealth quintiles. This suggests that promoting the consumption of blackjack leaves through behavior change interventions has the potential to increase calcium intake, particularly among poorer households, although the overall contribution to absorbable calcium may be limited by low bioavailability.

Additionally, the fortification of wheat flour with calcium could offer a cost-effective and scalable strategy, as calcium that is added as calcium carbonate through fortification is generally well absorbed, and wheat flour fortification programs have successfully improved the calcium intake in high-income countries [39]. Such an intervention would also benefit pregnant women by increasing their calcium intake before pregnancy, potentially reducing the risk of pre-eclampsia. However, lower bread consumption in the southern region, as well as in rural areas and among less wealthy households, may reduce its impact in these settings; nonetheless, even a modest consumption could provide additional absorbable calcium on top of the existing intakes from cassava leaves and roots and from drinking water, thereby contributing to an improved overall intake. In children who are 6–23 months of age, breastmilk contributed around one-fifth to the daily calcium intake; given its high calcium bioavailability (approximately 55–60% [12]), continued breastfeeding should be promoted.

Finally, factors such as infection, inflammation, and diarrhea, which are prevalent in this setting, may further compromise the calcium status by impairing intestinal absorption and increasing the losses of divalent ions, including calcium [41]. Therefore, interventions should consider not only increasing calcium intake but also improving the bioavailability of calcium from local diets and addressing the underlying health conditions.

This study has several strengths. It is nationally representative and includes the most vulnerable population groups, spanning across a wide age range and across both sexes of young children and adolescents. The large sample size allowed for residence stratification and zone-specific analyses, providing insight into regional differences. The dietary intake method was non-invasive, making it feasible to implement in a large national survey. However, several limitations should be considered. As with all dietary assessment methods, our calcium-specific FFQ relied on the participants’ or the participants’ caregivers’ ability to accurately recall the type and quantity of food that was consumed over the 7-day recall period, which may introduce a recall bias. The absence of a reliable biomarker for the calcium status limits the ability to validate the intake estimates and assess the physiological adequacy. Despite these limitations, the FFQ approach remains one of the most feasible methods for assessing the dietary calcium intake in a large population.

## 5. Conclusions

In conclusion, young children, adolescents, and nonpregnant and pregnant women in Madagascar face a high risk of inadequate calcium intake across all regions. Interventions are urgently needed to support the adequate consumption of calcium for growth, bone health, and maternal health. Promoting continued breastfeeding and the consumption of locally available calcium-rich foods, such as small fish consumed with bones and leafy green vegetables (e.g., blackjack and cassava leaves), should remain central strategies, particularly in rural and poorer populations. Given their high bioavailability, small fish represent an especially important dietary source of calcium. Calcium fortification, particularly of wheat flour, offers a complementary and scalable approach, with a greater potential impact in urban and wealthier populations. Together, these strategies—addressing both calcium intake and bioavailability—could contribute to reducing the burden of calcium deficiency in Madagascar.

## Figures and Tables

**Table 1:** Mean and median calcium intake and prevalence of inadequate intake in different population groups in Madagascar ^1^.

	Children (6–59 mo)	Adolescent Boys (10–19 y)	Adolescent Girls ^2^ (10–19 y)	Nonpregnant WRA ^2^ (15–49 y)	Pregnant Women (15–49 y)
*N*	2168	1543	1635	3398	283
Mean (95% CI) calcium intake (mg/d)	173 (162, 183)	296 (269, 322)	253 (237, 270)	281 (268, 295)	291 (261, 322)
Median (95% CI) calcium intake (mg/d)	156 (148, 164)	239 (213, 265)	203 (189, 217)	226 (209, 243)	234 (207, 260)
EAR for calcium (mg/d) [12]	260 (7–12 mo)	1100 (10–18 y)	1100 (10–18 y)	1100 (15–18 y)	1100 (14–18 y)
	500 (13–47 mo)	800 (19 y)	800 (19 y)	800 (19–49 y)	800 (>18 y)
	800 (48–59 mo)				
Risk of inadequate intake % (95% CI)	96.5 (95.1, 97.6)	97.9 (96.3, 98.8)	99.1 (98.5, 99.5)	97.1 (96.2, 97.8)	97.4 (94.2, 98.9)

^1^ The means, medians and percentages were weighted for the unequal probability of selection. The CI was calculated taking into account the complex sampling design. ^2^ The nonpregnant girls between 15 and 19 years old were counted in both categories, adolescent girls and nonpregnant WRA. EAR, estimated average requirement [12] and WRA, women of reproductive age.

**Table 2:** The estimated daily calcium intake in mg/day by various socio-demographic characteristics and population groups in Madagascar.

Characteristics	Children (6–59 mo)		Adolescents ^1^ (10–19 y)		Nonpregnant WRA ^1^ (15–49 y)	
	Mean (95% CI) ^2^	*p*	Mean (95% CI) ^2^	*p*	Mean (95% CI) ^2^	*p*
Gender		0.025		<0.001		-
Male	179 (164, 193)		296 (269, 322)		-	
Female	166 (155, 177)		253 (237, 270)		-	
Breastfeeding		-		-		0.006
Yes	-		-		299 (276, 322)	
No	-		-		274 (259, 290)	
Residence		0.260		<0.001		<0.001
Rural	171 (160, 183)		282 (260, 303)		288 (272, 304)	
Urban	180 (154, 205)		232 (196, 267)		253 (218, 289)	
Zone		0.003		<0.001		<0.001
North	188 (163, 213)		329 (281, 377)		317 (287, 347)	
Center	166 (149, 183)		225 (203, 247)		243 (223, 263)	
East	153 (126, 179)		244 (215, 273)		240 (210, 270)	
West	148 (116, 179)		274 (183, 366)		302 (212, 393)	
South	181 (164, 197)		324 (289, 359)		343 (317, 369)	
Wealth quintile ^3^		<0.001		<0.001		<0.001
Lowest	167 (150, 184)		302 (269, 335)		322 (293, 352)	
Second	160 (147, 173)		293 (262, 324)		296 (270, 321)	
Middle	158 (141, 175)		273 (243, 302)		280 (251, 310)	
Fourth	185 (161, 208)		262 (220, 304)		270 (240, 300)	
Highest	203 (171, 234)		232 (199, 265)		245 (216, 274)	
Household food security ^4^		<0.001		0.012		0.105
Severely insecure	171 (157, 185)		275 (252, 298)		289 (268, 309)	
Moderately insecure	157 (142, 171)		265 (237, 294)		271 (248, 294)	
Mildly insecure	172 (141, 203)		255 (219, 292)		267 (233, 301)	
Secure	199 (170, 227)		295 (248, 343)		291 (264, 318)	

^1^ The nonpregnant girls between 15 and 19 years old are counted in both categories, adolescents and nonpregnant WRA. WRA, women of reproductive age. ^2^ The means were weighted for the unequal probability of selection. The CI was calculated taking into account the complex sampling design. The sub-group differences were assessed using a one-way ANOVA. ^3^ assessed by the DHS Wealth Index. ^4^ assessed by the Food and Nutrition Technical Assistance Household Food Insecurity Access Scale.

## Data Availability

To obtain ethical clearance for the study, compliance with specific privacy standards was required. Accordingly, the deidentified data described in the manuscript, along with the codebook, and analytic code, may be made available upon request pending approval.

## Supplementary Materials

The following supporting information can be downloaded at https://www.mdpi.com/article/10.3390/nu18071041/s1. Table S1: The calcium content in calcium-rich foods used in the food frequency questionnaire; Table S2: the estimated average requirements for calcium and average drinking water and breastmilk intake by different populations and age groups; Table S3: the association of food groups that are included in the food frequency questionnaire with the total calcium intake using multiple linear regression analysis.

## Abbreviations

The following abbreviations are used in this manuscript:

AME	Adult male equivalent
EAR	Estimated average requirement
ENCM	Enquête nationale sur les carences en micronutriments à Madagascar
FFQ	Food frequency questionnaire
SUA	Supply utilization accounts
WRA	Women of reproductive age
