# Supplementary material for: Calcium Intake and Food Sources Among Children, Adolescents and Women in Madagascar: Results from a Nationally Representative Survey
Source: Nutrients. 2026 Mar 25;18(7):1041. doi: 10.3390/nu18071041 (PMC13074610; doi:10.3390/nu18071041)
Supplement: Supplementary file 1 [file nutrients-18-01041-s001.zip › nutrients-4178774-supplementary.pdf]

Supplementary Tables

Table S1. Calcium content in calcium-rich foods used in the food frequency questionnaire.

Food	Calcium (mg/100g)	Food composition table/reference
Large fresh fish, fresh crustaceans, fresh shrimp (bones/shell not eaten)	29	Madagascar [1]
Small fresh fish eaten entirely	550	Madagascar [1]
Large dried fish, dried shrimp (bones/shell not eaten)	63	Madagascar [1]
Small dried fish eaten entirely	2250	Madagascar [1]
Eggs	60	West Africa [2]
Milk	128	West Africa [2]
Infant cereals	50	Cerelac Nestlé, Magnin 2017 [3]
Infant formula	90	Malawi [4]
Boiled cassava leaves	289	West Africa [2]
Boiled blackjack leaves	800	Malawi [4]
Boiled cowpeas, lentils, Bambara beans	28	West Africa [2]
Boiled green beans (string beans)	40	West Africa [2]
Oranges	30	West Africa [2], Malawi [4]
Fresh sugarcane	13	Madagascar [1], Mozambique [5], Bettani 2024 [6]
Baguette and doughnuts	48	Malawi [4], Mozambique [5], West Africa [2]
Biscuits	67	West Africa [2]
Boiled cassava	32	West Africa [2]
Boiled cassava flour, boiled dried potatoes	26	West Africa [2]
Breastmilk	24	Allen 2025 [7]
Drinking water [1] (range)	0–71.5 mg/L	Analyzed at cluster level

[1] Measured with a semi-quantitative test on 1 water sample in each cluster.

Table S2. Estimated average requirements for calcium and average drinking water and breastmilk intake by different population and age groups.

Age group	Calcium (mg/day) [8]		Water (ml/day) [9] [a]		Breastmilk (ml/day) [10,11]
	Male	Female	Male	Female	
**Children**					
7–12 months [b]	260	260	600	600	680 [c], 550 [d]
13–47 months	500	500	900	900	550 [d]
48–59 months	800	800	1200	1200	-
**Adolescents & adults**					
10–13 years old	1100	1100	1800	1600	-
14–18 years old	1100	1100	2600	1800	-
19–49 years old	800	800	3000	2200	-
Breastfeeding women aged 14–18 years	-	1100	-	3100	-
Breastfeeding women aged 19–45 years	-	800	-	3100	-
Pregnant women aged 14–18 years	-	1100	-	2300	-
Pregnant women aged 19–45 years	-	800	-	2300	-

[a] Values for temperate climates. No reference available for tropical climates.
[b] Average requirements estimated as not established for this age group, adequate intake was used.
[c] Average intake for children 6-8.5 months of age [10].
[d] Average intake for children 9-23 months of age [11].

Table S3. Association of food groups included in the food frequency questionnaire with total calcium intake using multiple linear regression analysis [1]

**Food group**	**Children (6–59 mo) N=2157**		**Adolescents (10–19 y) [2] N=3158**		**Nonpregnant WRA (15–49 y) [2] N=3398**	
	**Coefficient (95% CI) [3]**	**p**	**Coefficient (95% CI) [3]**	**p**	**Coefficient (95% CI) [3]**	**p**
Intercept	5.57 (4.81, 6.33)	<0.001	5.55 (5.12,5.98)	<0.001	6.28 (5.96,6.61)	<0.001
Large fresh fish, fresh crustaceans, fresh shrimp (bones/shell not eaten)	0.48 (0.25, 0.72)	<0.001	0.23 (0.08,0.37)	0.002	0.16 (0.05,0.27)	0.004
Small fresh fish eaten entirely	1.72 (1.38, 2.05)	<0.001	0.87 (0.70,1.05)	<0.001	0.82 (0.67,0.98)	<0.001
Large dried fish, dried shrimp (bones/shell not eaten)	0.00 (-0.24, 0.24)	0.991	0.01 (-0.16,0.18)	0.934	-0.07 (-0.22,0.09)	0.396
Small dried fish eaten entirely	1.59 (1.25, 1.93)	<0.001	0.74 (0.55,0.94)	<0.001	0.84 (0.71,0.98)	<0.001
Eggs	0.33 (-0.19, 0.85)	0.214	0.16 (-0.10,0.42)	0.223	0.14 (-0.04,0.32)	0.132
Milk	2.10 (1.33, 2.87)	<0.001	0.90 (0.65,1.16)	<0.001	0.65 (0.45,0.85)	<0.001
Infant cereals	1.23 (0.50, 1.95)	0.001	-	-	-	-
Infant formula	4.86 (1.88, 7.83)	0.001	-	-	-	-
Boiled cassava leaves	1.94 (1.69, 2.19)	<0.001	1.29 (1.13,1.44)	<0.001	1.16 (1.02,1.30)	<0.001
Boiled blackjack leaves	2.63 (1.73, 3.53)	<0.001	1.80 (1.43,2.17)	<0.001	1.78 (1.42,2.14)	<0.001
Boiled cowpeas, lentils, Bambara beans	-0.06 (-0.25, 0.14)	0.567	0.03 (-0.14,0.19)	0.731	-0.02 (-0.15,0.10)	0.713
Boiled green beans (string beans)	-0.26 (-1.00, 0.47)	0.481	-0.01 (-0.35,0.33)	0.960	0.16 (-0.10,0.42)	0.236
Oranges	0.72 (0.45, 1.00)	<0.001	0.63 (0.47,0.80)	<0.001	0.42 (0.27,0.56)	<0.001
Fresh sugarcane	0.99 (0.71, 1.27)	<0.001	0.76 (0.60,0.91)	<0.001	0.54 (0.42,0.67)	<0.001
Baguette, doughnuts	0.57 (0.30, 0.83)	<0.001	0.13 (-0.02,0.28)	0.096	0.02 (-0.11,0.16)	0.731
Biscuits	0.08 (-0.15, 0.31)	0.499	0.12 (-0.04,0.29)	0.146	0.14 (-0.08,0.37)	0.219
Boiled cassava	0.45 (0.17, 0.72)	0.001	0.61 (0.43,0.78)	<0.001	0.49 (0.35,0.62)	<0.001
Boiled cassava flour, boiled dried potatoes	1.41 (0.52, 2.30)	0.002	0.30 (-0.18,0.78)	0.222	0.56 (0.28,0.84)	<0.001
Breastmilk	3.45 (2.97, 3.94)	<0.001	-	-	-	-
Drinking water [4]	0.03 (0.02, 0.04)	<0.001	0.03 (0.02, 0.03)	<0.001	0.02 (0.02,0.03)	<0.001

[1] Weighted multivariable linear regression analysis on Box-Cox transformed total calcium intake (mg/day) accounting for age, residence, zone and wealth quintile, and where applicable, sex.
[2] Nonpregnant girls between 15-19 years old are counted in both categories, adolescents and nonpregnant WRA. WRA, women of reproductive age.
[3] CI calculated taking into account the complex survey design.
[4] Calcium concentration measured with a semi-quantitative test on one water sample in each cluster.

References

1. François, P. *Budgets et Alimentation Des Menages Ruraux En 1962. Tome 2: Nutrition et Sociologie Alimentaire*; 1968;
2. Vincent, A.; Grande, F.; Compaoré, E.; et.al *FAO/INFOODS Food Composition Table for Western Africa (2019) User Guide & Condensed Food Composition Table*; Rome, 2020;
3. Magnin, M.; Stoll, B.; Voahangy, R.; Jeannot, E. Most Children Who Took Part in a Comprehensive Malnutrition Programme in Madagascar Reached and Maintained the Recovery Threshold. *Acta Paediatr.* **2017**, *106*, 960–966, doi:10.1111/apa.13796.
4. MAFOODS *Malawian Food Composition Table 2019*; Lilongwe, 2019;
5. Korkalo, L.; Hauta-alus, H.; Mutanen, M. *Food Composition Tables for Mozambique*; Helsinki, 2011;
6. Bettani, S.R.; Borges, M.T.M.R.; Soares, M.R.; Liska, G.R.; Rodrigues, C.E. da C. Relationship between the Mineral Content of Sugarcane and Its Genuine Derivative, Non-Centrifugal Raw Cane Sugar. *Food Res. Int.* **2024**, *192*, 114783, doi:10.1016/j.foodres.2024.114783.
7. Allen, L.H.; Islam, M.M.; Kac, G.; Michaelsen, K.F.; Moore, S.E.; Andersson, M.; Peerson, J.M.; Doel, A.M.; Dror, D.K.; Shahab-ferdows, S.; et al. Reference Values for Minerals in Human Milk : The Mothers , Infants and Lactation Quality (MILQ) Study. **2025**, *16*, doi:10.1016/j.advnut.2025.100431.
8. Institute of Medicine *Dietary Reference Intakes for Calcium and Vitamin D*; 2011;
9. Howard, G.; Bartram, J.; Williams, A.; Overbo, A.; Fuenta, D.; Geere, J. *Domestic Water Quantity, Service Level and Health*; Geneva, 2020;
10. Moore, S.E.; Devi, S.; Kurpad, A.; Peerson, J.M.; Christensen, S.H.; Islam, M.; Kac, G.; Michaelsen, K.F.; Silva, G.T.; Allen, L.H.; et al. Breast Milk Intake from 1 to 8 . 5 Months of Lactation in the Multisite Mothers , Infants and Lactation Quality (MILQ) Study. **2025**, *16*, doi:10.1016/j.advnut.2025.100456.
11. Dewey, K.G.; Brown, K.H. Update on Technical Issues Concerning Complementary Feeding of Young Children in Developing Countries and Implications for Intervention Programs. *Food Nutr. Bull.* **2003**, *24*, 5–28.
